# Ultrasound radiomics in personalized breast management: Current status and future prospects

**DOI:** 10.3389/fonc.2022.963612

**Published:** 2022-08-17

**Authors:** Jionghui Gu, Tian'an Jiang

**Affiliations:** ^1^Department of Ultrasound, The First Affiliated Hospital, College of Medicine, Zhejiang University, Hangzhou, China; ^2^Key Laboratory of Pulsed Power Translational Medicine of Zhejiang Province, Hangzhou, China; ^3^Zhejiang University Cancer Center, Hangzhou, China

**Keywords:** ultrasound, radiomics, breast, personalized medicine, artificial intelligence

## Abstract

Breast cancer is the most common cancer in women worldwide. Providing accurate and efficient diagnosis, risk stratification and timely adjustment of treatment strategies are essential steps in achieving precision medicine before, during and after treatment. Radiomics provides image information that cannot be recognized by the naked eye through deep mining of medical images. Several studies have shown that radiomics, as a second reader of medical images, can assist physicians not only in the detection and diagnosis of breast lesions but also in the assessment of risk stratification and prediction of treatment response. Recently, more and more studies have focused on the application of ultrasound radiomics in breast management. We summarized recent research advances in ultrasound radiomics for the diagnosis of benign and malignant breast lesions, prediction of molecular subtype, assessment of lymph node status, prediction of neoadjuvant chemotherapy response, and prediction of survival. In addition, we discuss the current challenges and future prospects of ultrasound radiomics.

## Introduction

Breast cancer (BC) has become the most commonly diagnosed malignancy among women worldwide, with approximately 2.3 millions new cases diagnosed and 680,000 deaths in 2020, which indicates that effective clinical strategies are urgently needed to manage BC patients (1). With the increasing advocacy of precision medicine, it is important to perform accurate and efficient diagnosis, risk stratification, and timely adjustment of treatment strategies before, during, and after treatment. Breast ultrasound (US) is one of the most important imaging technology and is used in clinical practice, which aims to monitor neoadjuvant chemotherapy (NAC) treatment and characterize breast lesions and axillary lymph nodes (2, 3). Various new US imaging techniques and quantitative analysis methods have been proposed, including US elastography and contrast-enhanced ultrasound (CEUS), to improve the sensitivity of conventional US and increase the accuracy of monitoring and prognostic prediction (4). However, it is difficult for radiologists to perform a comprehensive analysis of tumors with the information obtained by looking at various ultrasound images (5, 6). Radiologists face great challenges in achieving stable and reliable interpretation and efficacy prediction of such multi-modal US images.

New opportunities have emerged with the advent of radiomics, a technique for extracting high-throughput quantitative features from medical images In recent years, radiomics based on X-ray, US, magnetic resonance imaging (MRI) and positron emission tomography-computed tomography (PET-CT) has proved to be useful for extracting a large number of image features that cannot be observed with the naked eye (7–10). In some tasks, it matches or exceeds human perception (11, 12). Ultrasound has the characteristics of large data size, multiple data types, and frequent examination, which makes ultrasound radiomics uniquely advantageous in clinical applications. Therefore, the application of ultrasound radiomics in BC is being explored positively.

In this review, we aimed to briefly introduce ultrasound radiomics and summarize its potential clinical applications in the diagnosis of benign and malignant breast lesions, prediction of molecular staging, assessment of lymph node status, prediction of NAC response, and the prediction of survival. Moreover, we discuss the current challenges of ultrasound radiomics and how it can be more quickly applied to clinical practice, and then to achieve precise personalized medical management for BC patients based on US images and clinicopathological information.

## Workflows of radiomics

**Figure 1:**
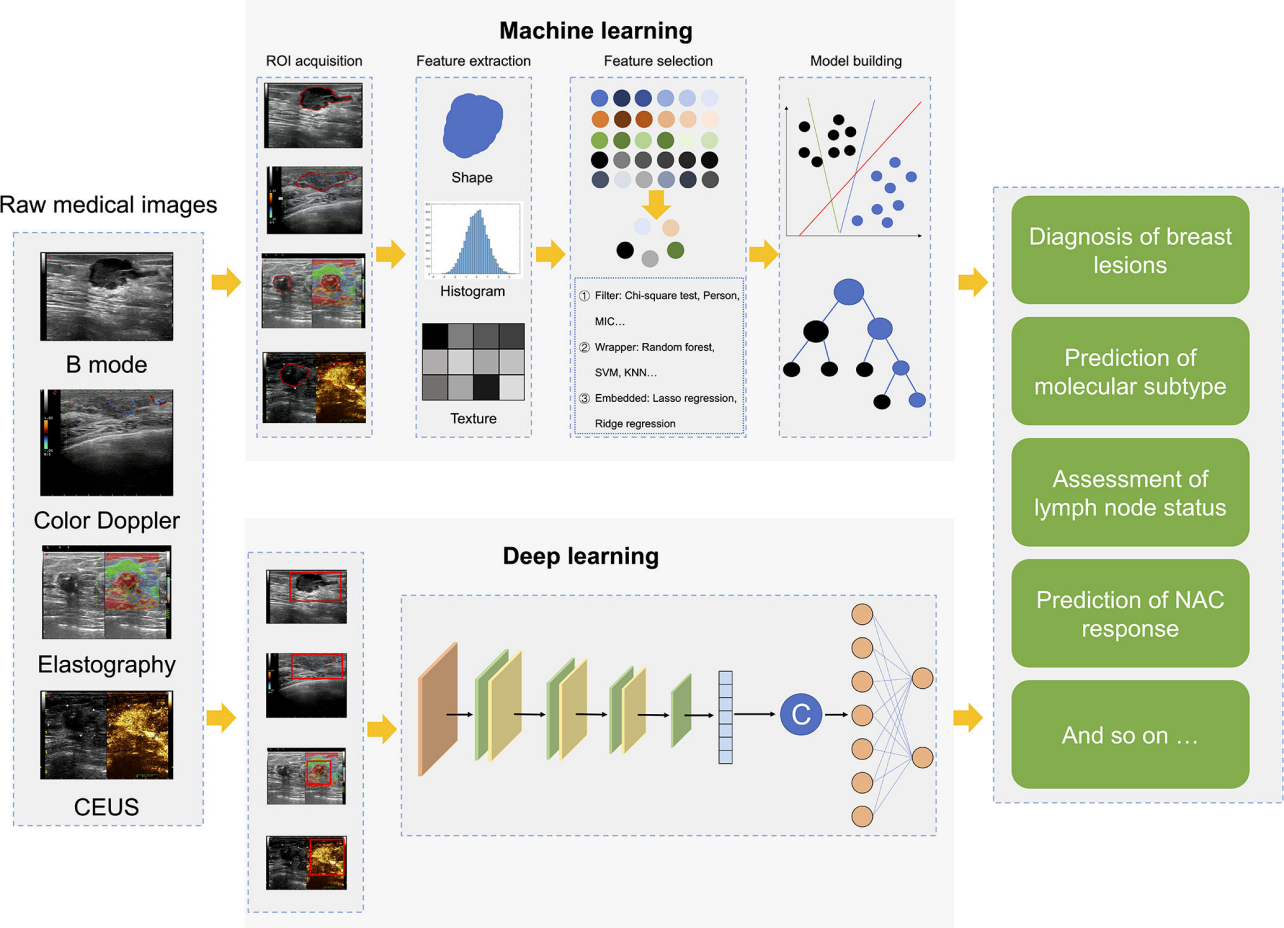
Radiomics workflows based on hand-crafted features or deep learning. CEUS, contrast-enhanced ultrasound; ROI, region of interest; MIC, mutual information and maximal information coefficient; SVM, support vector machine; KNN, k nearest neighbor; NAC, neoadjuvant chemotherapy.

Radiomics is an effective combination of big data analysis technology and medical images, which utilizes a large number of data characterization algorithms based on artificial intelligence to extract high-throughput quantitative image features from massive medical images and build a data information bank (13, 14). Then, radiomics performs deep learning analysis and information mining from these quantified image features and link them with clinical macroscopic information and pathological and/or genetic microscopic information, which holds potential in disease detection, diagnosis, prognosis, and treatment (13). At present, radiomics strategies mainly include two methods (13, 15). One is the classic approach based on extracting pre-designed (also referred to as hand-crafted or engineered) features using conventional machine learning (ML) (**Figure 1**). The other is the recently developed approach based on deep learning (DL), it can autonomously learn and extract complex and abstract features related to disease from a large number of medical images by constructing a multi-layer neural network, without any prior design (**Figure 1**).

The radiomics process based on engineered features can be divided into five steps: 1) Medical image acquisition, which can be various types of medical images, such as X-ray, computed tomography (CT), MRI, PET-CT, US, or even images of H&E-stained biopsy sections. 2) Region of interest (ROI) segmentation, which is to extract only the information related to the lesion. The current segmentation of ROI mainly includes manual segmentation, semi-automatic segmentation and automatic segmentation. Different segmentation algorithms have their applicable scope and conditions. There is no universal segmentation algorithm with high recognition yet. 3) Feature extraction: Radiomics features are extracted from ROI, including signal intensity, shape, size, and first-order, second-order and higher-order texture features. 4) Feature selection: Although radiomics extracts many features, some features are spurious and redundant for a specific task. Therefore, it is necessary to select features with good repeatability, high stability and independence according to feature selection methods, which is conducive to the construction of robust models. At present, the main methods include least absolute shrinkage and selection operator (LASSO), recursive feature elimination, principal component analysis, and max-relevance and min-redundancy, etc. 5) Model building and validation: This mainly refers to model building and testing independent samples, which can be done by a variety of methods, from statistics to advanced machine learning strategies. The common methods include linear regression, logistic regression, support vector machine, random forest, Cox regression, artificial neural network and so on.

In recent years, with the exponential increase of GPU computing power and the development of medical big data, DL has become one of the most popular analysis methods in radiomics (16). DL-based radiomics (DLR) is an end-to-end model that does not require human involvement. The feature extraction and analysis parts of DLR are coupled. While hand-crafted feature-based radiomics requires pre-determination along with expert knowledge, DLR does not require the preparation of pre-defined features, which reduces the subjectivity and uncertainty of hand-crafted feature design and selection.

## Radiomics in the ultrasound

Compared with other imaging techniques, US has the advantages of simple, no radiation and real-time, and is one of the most important methods for monitoring breast lesions. In recent years, with the continuous development of ultrasound instruments, various new ultrasound techniques such as color doppler imaging, contrast-enhanced ultrasound (CEUS) and US elastography have also been used as complementary techniques for breast examination. Radiologists’ demand for efficient and objective assessment of US images in routine clinical work is increasing, and AI-assisted ultrasound image analysis has attracted attention.

The traditional radiomics based on feature engineering requires manual segmentation of target regions and manual definition of features on images. However, it is difficult to perform manual segmentation of US images due to low resolution and vague boundary definition. Additionally, the repeatability of US examinations is easily affected by different operators, patients and instruments. Therefore, the application of machine learning based on feature engineering in US image analysis has certain limitations. However, the DLR approach supports a simple end-to-end training or learning process that can create a fully automated workflow. Moreover, deep learning networks can learn specific features from the data itself. Therefore, DLR can better enable the analytical processing of US images and improve the dependence of US images on various operators, patients and machines. DLR is expected to achieve robust and scalable ultrasound radiomics models to assist in disease detection, diagnosis, prognosis, and treatment.

## Ultrasound radiomics in the breast diagnosis

Although ultrasonography is the one of most common imaging technique used to detect and distinguish benign and malignant breast lesions, it is difficult to accurately and stably identify some lesions with the naked eye. Recently, many studies have explored the potential of ultrasound radiomics to aid in the detection and differentiation of lesions (9, 17–20) (**Table 1**).

**Table 1:** Summary of ultrasound radiomics studies in breast diagnosis.

Study	Task	Data size	Imaging data	Radiomics results
Fleury et al. (17) 2020	benign vs malignant	207 lesions	2D-US	AUC: 0.817
Li et al. (18) 2021	benign vs malignant	256 lesions	2D-US	AUC: 0.943
Romeo et al. (9) 2021	benign vs malignant	201 lesions	2D-US	AUC: 0.820
Shen et al. (21) 2021	benign vs malignant	143203	2D-US + Color Doppler	AUC: 0.962
Fujioka et al. (22) 2020	benign vs malignant	377 lesions	SWE-US	AUC: 0.898
Ciritsis et al. (20) 2019	Task A: BI-RADS 2 vs BI-RADS 3-5; Task B: BI-RADS 2-3 vs BI-RADS 4-5	582 lesions	2D-US + radiological report	ACC: 0.930 for task A; ACC: 0.953 for task B
Mango et al. (19) 2020	benign vs malignant	900 lesions	2D-US	AUC: 0.870
Moustafa et al. (23) 2020	benign vs malignant	159 lesions	2D-US + Color Doppler	AUC: 0.958
Fujioka et al. (24) 2019	benign vs malignant	360 lesions	2D-US	AUC: 0.913
Dong et al. (25) 2021	benign vs malignant	367 lesions	2D-US	AUC: 0.899 with coarse ROIs AUC: 0.869 with fine ROIs
Qian et al. (26) 2021	benign vs malignant	873 lesions	2D-US + Color Doppler + elastography	AUC: 0.922 (2D-US + Color Doppler) AUC: 0.955 (2D-US + Color Doppler + elastography)
Zhang et al. (27) 2021	benign vs malignant	1311 lesions	2D-US	AUC: 0.846 PPV:9.3% for BI-RADS 4A
Chen et al. (28) 2021	benign vs malignant	221 lesions	CEUS	ACC: 0.863
Jiang et al. (29) 2021	benign vs malignant	401 lesions	2D-US + SWE	AUC: 0.920
Zhang et al. (30) 2019	benign vs malignant	227 lesions	2D-US + SWE	AUC: 0.961
Misra et al. (31) 2022	benign vs malignant	85 lesions	2D-US + SE	ACC: 0.900
Zhang et al. (32)2020	benign vs malignant	291 lesions	2D-US + SWE	ACC: 1.000

US: ultrasound, SWE: shear wave elastography, SE: strain elastography, AUC: area under the curve, ACC: accuracy, PPV: positive predictive value

Earlier, Fujioka et al. (24) began to use the DLR model based on US images to identify benign and malignant breast lesions. This study confirmed that the DLR model had equal or better diagnostic performance compared to radiologists on a test dataset with 120 breast lesions (AUC = 0.913 *vs* 0.728-0.845, p = 0.01-0.14). Subsequently, several studies have shown that ultrasound radiomics based on 2D-US images has good performance in identifying benign from malignant breast lesion, with AUCs ranging from 0.817-0.943 (9, 17–19). Additionally, studies have shown that the classification performance of the AI model may be affected by adjusting the ROI as different inputs of the model. Dong et al. (25) proposed that the performance of the DLR model with coarse ROI is slightly better than the DLR model with fine ROI. Therefore, we can conclude that peripheral tissue is also an important factor in the classification of breast lesions.

Since breast ultrasonography has a high rate of false positives (FP), how to reduce the rate of FP with artificial intelligence (AI) has attracted extensive attention by researchers. Chen et al. (21) established an AI model with 288,767 US examinations in a retrospective study and demonstrated that with the assistance of AI, radiologists reduced the FP rate by 37.3% and unnecessary biopsies by 27.8% without sacrificing sensitivity. And several other studies have also confirmed this finding (33, 34). Recent studies have challenged the use of ultrasound radiomics for specific breast lesions that are difficult to diagnose in clinical practice, particularly for BI-RADS 4A lesions. Niu et al. (35) analyzed 206 patients with a US score of BI-RADS 4A and concluded that AI can reveal more subtle differences associated with benign-malignant differentiation in BI-RADS 4A lesions compared to the naked eye. Thus, with the morphological and textural information provided by AI, physicians can make more accurate judgments about such atypical lesions. In addition, a study by Zhang et al. (27) confirmed a positive predictive value was 9.3% when using the AI model to analyze BI-RADS 4A lesions. Although this result was not significant, it was superior to radiologists.

Studies have shown that radiomic features extracted from multimodal US images can improve the ability of lesion diagnosis. A recent study by Zhan et al. (30) showed that dual-mode image features from 2D and shear wave elastography (SWE) achieved accurate differentiation for malignant and benign breast tumors with an AUC of 0.961, which employed a framework for feature learning and classification with the deep polynomial network. Several studies have further confirmed the superior performance of ultrasound radiomics based on bimodal US images in classifying over quantitative elastography parameters (22, 29, 31, 32, 36, 37). As known, the blood supply characteristics of breast masses are important features to determine the malignancy of the lesion. Moustafa et al. (23) extracted radiomics features from 2D-US and color doppler images, respectively, to establish DLR models to help determine the possibility of malignant. CEUS can provide more detailed blood supply characteristics, which can be used to establish an AI model for the differential diagnosis of breast cancer (28). The interpretability and clinical applicability of the DLR model have always been two major challenges in the field of AI. Notably, an interpretable and clinically applicable DLR system was recently proposed and validated by Qian et al. (26). The study used 10,815 and 912 multi-modal (B mode, color doppler and elastography) multi-view (transverse and longitudinal) breast US images for training and prospective testing, respectively, and had an AUC of 0.955 finally. Such a clinically applicable AI system may be incorporated into future breast cancer US screening, as well as workflows that support ancillary or secondary readings.

## Ultrasound radiomics in the evaluation of molecular subtype

BC is a highly heterogeneous tumor, and the molecular expression status is one of the key factors indicating the prognosis and guiding the choice of treatment options. At present, molecular subtypes of BC are mainly determined by genetic or immunohistochemistry analysis. However, there are false negatives for biopsy results of individual tissues. The ultrasound radiomics is based on the assumption that microstructural discrepancies in different molecular subtypes of breast cancer result in different gray-scale patterns, margins, or any other features on US images that can be identified by AI models. Currently, researchers are attempting to use ultrasound imaging histology to non-invasively and comprehensively analyze the molecular status of the entire tumor tissue to provide personalized management for BC patients (**Table 2**).

**Table 2:** Summary of ultrasound radiomics studies in classifying breast cancer subtypes.

Study	Task	Data size	Imaging data	Radiomics results
Jiang et al. (38) 2021	assessment of four breast cancer molecular subtypes: luminal A, luminal B, HER2+, triple-negative	2120 lesions	2D-US	ACC: form 0.8007 to 0.9702 for the test cohort A; and 0.8794 to 0.9883 for the test cohort B for each sub-category
Guo et al. (39) 2018	distinguish between HR+/HER2- and triple-negative	215 lesions	2D-US	AUC: 0.760
Wu et al. (40) 2021	predicting the expression of ER, PR, HER2, Ki67, P16, and P53	116 lesions	2D-US	AUC: ER (0.940 and 0.840), PR (0.900 and 0.780), HER2 (0.940 and 0.740), Ki67 (0.950 and 0.860), P16 (0.960 and 0.780), and P53 (0.95 and 0.74) in training and test cohort, respectively.
Cui et al. (41) 2021	predicting the expression of Ki67 and P53	263 lesions	2D-US	AUC: 0.780 for Ki67; 0.710 for P53
Li et al. (42) 2021	predicting the expression of Ki67 and HER2	252 lesions	2D-US	AUC: 0.680 for Ki67; 0.651 for HER2

US, ultrasound; HER2, human epidermal growth factor receptor 2; HR, hormone receptor; ER, estrogen receptor; PR, progesterone receptor; AUC, area under the curve; ACC, accuracy.

Studies have shown that ultrasound radiomics is expected to be a new imaging label for identifying molecular subtypes (HER2+, triple-negative, Luminal A and Luminal B) of BC patients because of its good performance (38, 39). In addition, Jiang et al. (38) confirmed that the DLR model could distinguish the luminal type and non-luminal type satisfactorily with AUCs of 0.87 and 0.83 in two independent test cohort. However, Wu et al. (40) extracted quantitative radiomics features of tumors in raw US images and showed a general performance in predicting molecular biomarker expression. The radiomics models showed predictive performance with AUC greater than 0.7 in the test cohort, and the AUCs are 0.84, 0.78, 0.74, 0.86, 0.78, and 0.74 for ER, PR, HER2, Ki67, p16, and p53, respectively. The treatment of triple-negative BC has been a challenge due to the absence of effective drugs for specific molecular targets. Whereas the expression of ki67 is a prognostic indicator and p53 is considered a tumor suppressor. Cui et a.l (41) and Li et al. (42) found that ultrasound radiomics models enabled preoperative classification of ki67 and p53 status. Furthermore, it is noteworthy that recent studies have shown that ultrasound radiomics features are not only a potential imaging biomarker for disease-free survival risk stratification, but also can predict the risk of postoperative recurrence in patients with triple-negative BC (43, 44). At present, the ultrasound radiomics in predicting molecular subtype and survival recurrence of BC needs further research.

## Ultrasound radiomics in the assessment of lymph node status

Accurate identification of axillary lymph node (ALN) status is important in determining tumor stage, developing appropriate axillary treatment plans, and predicting prognosis for BC patients with or without NAC treatment (2, 3). Sentinel lymph node (SLN) biopsy and axillary lymph node dissection (ALND) are two main methods for determining ALN status. It is worth mentioning that there are varying degrees of complications with both sentinel lymph node dissection and ALND (45, 46). Thus, the development of noninvasive biomarkers to identify ALN status is of great significance for the accurate management of BC patients. At present, researchers are challenging the radiomics approach based on primary breast tumors on US images in predicting the status of ALN and SLN (**Table 3**).

**Table 3:** Summary of ultrasound radiomics studies in predicting axillary lymph node status.

Study	Task	Data size	Imaging data	Radiomics results
Lee et al. (47) 2021	Predicting ALN metastasis	496 patients	2D-US	AUC: 0.810
Qiu et al. (48) 2020	Predicting ALN metastasis	196 patients	2D-US	AUC: 0.759
Zhou et al. (49) 2021	Predicting ALN metastasis	192 patients	2D-US	AUC: 0.650
Yu et al. (50) 2019	Predicting ALN metastasis	426 patients	2D-US	AUC: 0.810
Guo et al. (51) 2020	Predicting SLN metastasis and NSLN metastasis	937 patients	2D-US	AUC: 0.848 for SLN metastasis; AUC: 0.812 for NSLN metastasis
Lee et al. (52) 2021	Predicting ALN metastasis	153 patients	2D-US	AUC: 0.805
Sun et al. (53) 2020	Predicting ALN metastasis	479 patients	2D-US	AUC: 0.950
Jiang et al. (54)2021	Predicting ALN burden	433 patients	2D-US+SWE	C-index: 0.817 for N0 and N+(≥ 1) C-index: 0.810 for N+(1-2) and N+(≥ 3)
Zheng et al. (55) 2020	Predicting ALN metastasis	584 patients	2D-US+SWE	AUC: 0.905
Gao et al. (56) 2021	Predicting ALN burden	343 patients	2D-US	AUC: 0.733 for N+(<3) and N+(≥ 3)

US, ultrasound; SWE, shear wave elastography; ALN, axillary lymph node; SLN, sentinel lymph node; NSLN, non-sentinel lymph node; AUC, area under the curve

The majority of the earliest studies using ultrasound radiomics to predict lymph node status were based on 2D grayscale US images. Several studies have confirmed that DLR combined with clinicopathological features has a satisfactory performance in predicting ALN metastasis, with an AUC between 0.75 and 0.85 (47–50). Guo et al. (51) proposed a DLR ultrasonography (DLRU) model for comprehensive evaluation of SLN and non-sentinel lymph node (NSLN) status. And DLRU achieved a sensitivity of 98.4% in identifying SLN+ and 98.4%in identifying NLSN+. In addition, Lee et al. (52) innovatively explored the performance of peritumoral region combined with tumor region in predicting lymph node metastasis (LNM) with method of ultrasound radiomics. They found that DLR model with 3mm thick peritumoral tissue tumor area had the best predictive performance, achieving an accuracy of 81.05% (124/153). Therefore, combining tumor and peri-tumor tissues contributes to the prediction of LNM, which is consistent with the results of previous study (53). SWE is an elastographic technique that integrates B-mode US with a color-coded map to allow better characterization of breast lesions. Jiang et al. (54) developed and validated a nomogram containing radiomics features of SWE for assessing the risk level of LNM in early BC, then the result confirmed that ultrasound radiomics model showed good predictive power for LNM risk staging in early-stage BC patients, which can provide incremental information for decision making. Moreover, recent studies have shown that clinical characteristics combined with DLR model based on multimodal US images (B mode and SWE) can provide a noninvasive and practical tool for preoperative prediction of ALN status, and achieve an AUC of 0.905 in the test cohort (55). Compared with the DLR model based on grayscale US images alone, the performance of the DLR model based on multimodal US images for tumor load of ALN achieved a significant improvement (56). As clinical practice proposes greater demands on precision treatment, studies with larger data size and more multimodal fusion are needed to confirm the validity of the DLR model.

## Ultrasound radiomics in the prediction of NAC response

NAC has become one of the most important treatment methods for BC patients. Normally, if the efficacy of NAC is unresponsive or unsatisfactory, further treatment should be changed accordingly. Therefore, early discontinuation of ineffective treatment or adjustment of treatment strategy is essential to avoid unnecessary toxicities and optimize overall benefits. However, owing to the heterogeneity and complexity of the tumor, individual responses of BC patients to NAC exhibit vast differences and tumors and axillary response to NAC are not parallel (57–60). Histopathological examination of surgical specimens is the gold standard for evaluating response and can only be performed after NAC treatment. Accurate assessment and prediction of response are of particular significance for the precise management of breast cancer patients who underwent NAC. Although MRI is currently the most important method for assessing NAC response (61–63), it still cannot predict pathologic complete response (PCR) with sufficient accuracy (64). MRI is not suitable for frequent monitoring during NAC treatment due to its high cost and time-consuming. Ultrasound is the most suitable examination method to be used repeatedly in the process of NAC. Several studies have shown that DLR based on US images has good performance in predicting the efficacy of NAC for BC patients (**Table 4**).

**Table 4:** Summary of ultrasound radiomics studies in predicting response of NAC.

Study	Task	Data size	Imaging data	Radiomics results
Quiaoit et al. (65) 2020	Predicting the response to NAC before surgery	59 patients	2D-US	AUC: 0.870
DiCenzo et al. (66) 2020	Predicting the response to NAC before treatment	82 patients	2D-US	ACC: 0.870
Sannachi et al. (67) 2019	Predicting the response to NAC	100 patients	2D-US	ACC: 0.780 at 1 week after the start of treatment ACC: 0.900 at 4 weeks after the start of treatment ACC: 0.920 at 8 week after the start of treatment
Jiang et al. (68) 2021	Predicting the response to NAC before surgery	592 patients	2D-US	AUC: 0.940
Byra et al. (69) 2021	Predicting the response to NAC	38 patients	2D-US	AUC: 0.844 (Pre NAC) AUC: 0.827 (after first course of NAC) AUC: 0.828 (after second course of NAC)
Gu et al. (70) 2021	Predicting the response to NAC	168 patients	2D-US	AUC: 0.812 (after second course of NAC) AUC: 0.937 (after fourth course of NAC)

NAC: neoadjuvant chemotherapy; US, ultrasound; AUC, area under the curve; ACC, accuracy.

Quiaoit et al. (65) attempted to explore the performance of quantitative ultrasound radiomics in monitoring the response to NAC on a dataset of 59 patients, and the results were generally consistent with those of other previous studies (66, 67, 71). The usefulness of quantitative ultrasound radiomics for NAC response assessment remained relatively limited. Recently, the emergence of DLR has greatly enhanced the image analysis capabilities of radiomics, which relies on deep neural network and data-driven learning to achieve automatic feature extraction and is promising in US images analysis. Jiang et al. (68) proposed an integrated ultrasound radiomics model based on a multicenter dataset of 592 individuals that combined deep learning and machine learning to predict PCR to NAC for BC patients. The deep learning radiomics nomogram model achieved an AUC of 0.94 in the validation cohort, with a significant improvement in predictive accuracy compared to two radiologists (p < 0.01). In addition to assessing the tumor status of patients at the end of NAC, predicting response early in NAC appears critical for early treatment change and avoiding unnecessary treatment. Byra et al. (69) and Gu et al. (70) proposed the Siamese convolutional neural network for predicting response at an early stage of NAC and achieved accurate and personalized prediction. Gu et al. also developed a deep learning radiomics pipeline using cascading models constructed at different courses of NAC treatment. Although, various studies have confirmed that the ultrasound radiomics can provide physicians with a valid and feasible tool to predict the response to NAC and determine further personalized treatment protocols. However, no large clinical trial has yet shown that ultrasound radiomics predictions can fully determine whether a patient should be discontinued from NAC. Clinicians must consider treatment strategies in combination with various resources and patients’ demands.

## Ultrasound radiomics and personalized management of BC

The personalized treatment plan for BC patients includes the timing and specific implementation of surgery, the timing and protocol of radiotherapy and chemotherapy, and other treatment strategies, all of which require comprehensive consideration of molecular subtypes, lymph node status, the efficacy of neoadjuvant therapy and other factors. However, BC is a heterogeneous disease with a high degree of diversity in biochemistry, histopathology and morphology, all of which affect treatment and clinical outcomes. In addition, most gold standards need to be obtained after surgery. Therefore, preoperative noninvasive assessment and prediction is the most important clinical issue in the realization of personalized management of BC patients, which has not been addressed by imaging methods at present. Ultrasound radiomics aims to extract a large number of high-throughput features to obtain more useful information about tissue lesions and treatment response information for personalized medicine. The solution by ultrasound radiomics is highly expected.

## Future challenges

Ultrasound radiomics transforms medical images into high-dimensional quantitative data, which help physicians understand the characteristics of tumor phenotypes (including the macroscopic phenotype of the tumor, and the cellular and molecular characteristics of the tumor tissue), and achieved impressive results in both diagnosis and prediction (13, 72). In addition, ultrasound radiomics, as a complement to biopsy analysis, can simultaneously assess the tumor microenvironment, characterize tumor spatial heterogeneity, and assess disease progression longitudinally with the advantage of non-invasive. However, it is still a long way to transfer ultrasound radiomics from scientific research to clinical practice, given some of the current limitations and challenges. First, ultrasound with handheld technology lacks reproducibility compared to other techniques such as mammography or MRI. Compared with radiomics based on ML, DLR can overcome this drawback to a certain extent. However, most of the previous studies were small sample single-center retrospective studies, which leads to the robustness of ultrasound radiomics models is not stable enough. Future internationalized multi-center data with larger sample sizes are needed to validate and improve the robustness of the models. In addition, due to the differences in imaging acquisition and the diversity of reconstruction algorithms, an exhaustive data management and coordination process is needed to obtain multi-center data. Second, there is a lack of effective methods to fuse multi-modal US data (such as B mode, color doppler, CEUS, and elastography) to perform a multi-scale and all-around assessment of tumor biological behavior (73). Finally, DLR is a “black box” technology, that lacks transparency and biological interpretability for algorithms (74). Therefore, how correlating DLR image features with biological information, and quantifying the key molecular information in the development of BC using tumor images, which are major challenges for future research. We believe this is important because radiomics plays a supporting role in the foreseeable future by providing physicians with more interpretative and understandable information.

Additionally, multi-omics studies have become a hot topic for characterizing the molecular biology of tumors, including genomics, transcriptomics, proteomics, and metabolomics (72, 75). Thus, multi-omics studies are accelerating BC research and making precision medicine possible. In the future, ultrasound radiomics should be combined with clinical data and microscopic genetic data to develop multi-omics studies, which may accelerate BC research in precision diagnosis, decision making and prediction. Although most DLR is still in the technology development stage, the development of genomics and deep learning technologies may facilitate the extraction of deep features and explore new possibilities in BC radiomics or radio-genomics.

## Conclusion

In conclusion, radiomics has emerged rapidly as one of the most interesting research topics in breast ultrasonography, with promising results for the clinical management of BC. This article has outlined the application of ultrasound radiomics in the clinical practice for the management of BC patients, including the diagnosis of benign and malignant lesions, prediction of molecular staging, assessment of lymph node status, prediction of NAC response and prediction of survival. Ultrasound radiomics is a promising tool for personalized precision medicine by virtue of its noninvasive nature. We also identify the limitations of radiomics that currently hinder its translation into clinical practice and strategies to overcome these limitations. In the future, the establishment of multi-omics studies including radiomics will hopefully connect the information extracted from breast US images to the tumor microenvironment, and provide precise and personalized treatment decisions for BC patients.

## Author contributions

JG and TJ, conception and design the study. JG and TJ, medical support and manuscript correction. JG, manuscript writing. TJ, expert guidance and manuscript review. All authors, final approval of the manuscript. All authors contributed to the article and approved the submitted version.

## Funding

This study was supported by the Development Project of National Major Scientific Research Instrument (82027803); National Natural Science Foundation of China (81971623); Key Project of Natural Science Foundation of Zhejiang Province (LZ20H180001); Zhejiang Provincial Association Project for Mathematical Medicine (LSY19H180015).

## Conflict of interest

The authors declare that the research was conducted in the absence of any commercial or financial relationships that could be construed as a potential conflict of interest.

## Publisher’s note

All claims expressed in this article are solely those of the authors and do not necessarily represent those of their affiliated organizations, or those of the publisher, the editors and the reviewers. Any product that may be evaluated in this article, or claim that may be made by its manufacturer, is not guaranteed or endorsed by the publisher.
